# The a subunit isoforms of vacuolar-type proton ATPase exhibit differential distribution in mouse perigastrulation embryos

**DOI:** 10.1038/s41598-022-18002-4

**Published:** 2022-08-10

**Authors:** Ge-Hong Sun-Wada, Yoh Wada

**Affiliations:** 1 Faculty of Pharmaceutical Sciences, Doshisha Women’s College of Liberal Arts, Kohdo, Kyotanabe, Kyoto 610-0395 Japan; 2 Division of Biological Science, Institute of Scientific and Industrial Research, Osaka University, 8-1 Mihogaoka, Ibaraki, Osaka 567-0047 Japan

**Keywords:** Cell biology, Developmental biology

## Abstract

Vacuolar-type H^+^-ATPases (V-ATPases) are large multi-subunit complexes that play critical roles in the acidification of a variety of intracellular or extracellular compartments. Mammalian cells contain four isoforms of the membrane integral subunit a (a1–a4); these isoforms contain the information necessary to target the enzyme to different cellular destinations. They are also involved in regulating the efficiency of ATP hydrolysis and proton transport. Previously, we showed that early embryogenesis requires V-ATPase function, and the luminal acidic endocytic and lysosomal compartments in the visceral endoderm of mouse embryos at the pre-gastrulation stage (E6.5) are essential for both nutrition and signal transduction during early embryogenesis. In this study, we examined the expression and distribution of a subunit isoforms in mouse embryos at E6.5. We found that all four isoforms expressed and exhibited differential distribution in the E6.5 embryo. At this developmental stage, the embryos establish highly elaborate endocytic compartments called apical vacuoles, on which the a3 isoform specifically accumulated.

## Introduction

The luminal acidification of intracellular and extracellular compartments is intimately related to diverse cellular activities, including proper vesicle trafficking at various developmental stages^1,2^. The acidification of intracellular organelles, including lysosomes, endosomes, and the Golgi apparatus, is established by the function of vacuolar-type H^+^-ATPases (V-ATPase). The same enzyme is also found in the plasma membranes of specialized cells such as osteoclasts, and cells of the kidney, inner ear, olfactory, and male reproductive tract epithelium^3,4^. Thus, according to the cellular context, V-ATPases can be localized to different organelles and membranes, exerting their specific functions.

V-ATPase consists of two major functional sections: V_1_ and V_0_. Cytosolic V_1_ has eight subunits (denoted as A–H) and contains three catalytic sites for ATP hydrolysis formed by subunits A and B. The membrane integral V_0_ section mediates proton translocation across membranes. The V_0_ section comprises up to six subunits, including a, d, and e, and the proteolipids c, c′, and c′′^5,6^. The mammalian V_0_ section contains two accessory proteins, ap1/Ac45 and ap2/(pro)renin receptor^7,8^. We have shown that the subunits of mammalian V-ATPase have several isoforms that are encoded by distinct loci. These subunit isoforms are, at least in part, responsible for specific functions of diverse tissues^9^.

In particular, the localization of V-ATPase to specific membranes within the cell is controlled by the 100-kDa subunit a of the V_0_ section. In the yeast *Saccharomyces cerevisiae*, two a subunit isoforms are encoded by *Vph1* and *Stv1* loci, and the former is localized to the vacuole, whereas the latter is Golgi-resident. This distinctive subcellular localization is defined by their interaction with specific phospholipids^10^. In mammals, four genes encoding a subunit isoforms (a1–a4) are present in the genome^11^. Isoform a4 is predominantly expressed in the plasma membrane of renal intercalated cells^12–14^, and epididymal clear cells^15^, whereas other isoforms (a1, a2, a3) are expressed ubiquitously in the tissues examined so far^16,17^. Isoform a3 is a component of the osteoclast plasma membrane enzyme^16,18–20^. Isoform a1 localizes to presynaptic nerve terminals, whereas a2 is mainly localized in the endosomes^3,21^. However, the physiological relevance of each a subunit isoform is still not fully understood.

We have shown that early embryogenesis requires V-ATPase function, particularly by establishing and maintaining apico-basolateral cell polarity in the embryonic epithelium^22^. Genetic inactivation of V-ATPase function results in loss of cell polarity in the visceral endoderm (VE)^22^, which is an essential tissue responsible for nutrient and waste exchange, as well as for active regulation of multiple signalling pathways guiding early development^23^. VE cells contain a characteristic large intracellular organelle called the apical vacuole (AV), exhibiting lysosomal characteristics. Dysfunctions in the intracellular vesicle trafficking pathway to the AV cause defects in early embryonic development, implying that the nutritional and signalling functions of VE are highly dependent on the endocytic organelles^24–28^.

These previous studies have shown that early embryos are equipped with highly elaborate endomembrane systems that enable the embryos to execute both autonomous developmental programs and interactions with maternal tissues. However, our knowledge of the precise roles of V-ATPase and intra- and extracellular acidification during early embryogenesis is still limited. In this study, we examined the expression and distribution of a subunit isoforms in mouse embryos at E6.5, a stage around the time of gastrulation. We found that a subunit isoforms were differentially localized in mouse early embryonic tissues.

## Materials and methods

### Antibodies and animals

Rabbit polyclonal or chick monoclonal antibodies raised against a subunit isoforms have been described previously^12,16,29^. In brief, the rabbit anti-mouse a1 subunit affinity purified antibodies were used at a dilution of 1/100^16^; the chick anti-a2 (OA560, clone 1-26-1) was diluted at 1/50^29^; the rabbit anti-mouse a3 subunit affinity purified antibodies were used at 1/100^16^, and the rabbit anti-mouse a4 subunit affinity purified antibodies were used at 1/500^12^. The specificities and titres of these antibodies have also been validated in recently publications^19,30–32^. The rat anti-lamp2 monoclonal antibody GL2A7-c was obtained from DSHB (Univ. Iowa). The secondary antibodies used were fluorescein isothiocyanate (FITC) -conjugated donkey anti-rat IgG antibodies and Cy3-conjugated donkey anti-rabbit IgG or chick IgY antibodies (Jackson ImmunoResearch, USA). All animal procedures were approved by the Committees of Institute of Scientific and Industrial Research (ISIR), Osaka University, and Doshisha Women’s College of Liberal Arts (DWCLA) and performed in accordance with institutional and national guidelines. In addition, all the animal studies were in compliance with ARRIVE guidelines. ICR mice were purchased from Japan SLC. The mice were provided with food and water ad libitum.

### *Whole-mount *in situ* hybridization*

Digoxigenin (DIG)-11-UTP-labeled single-stranded RNA probes were synthesized using a DIG RNA labelling mix (Roche, Switzerland) and the corresponding T3 or T7 RNA polymerase according to the manufacturer's instructions. The 899, 780, 727, and 460-bp fragments derived from the 3′ untranslated regions of a1, a2, a3, and a4, respectively, were amplified by PCR using the primer set (Table 1) and genomic DNA or BAC clones as templates. The PCR products were cloned into the pGEM11 vector, and their sequences were verified. These plasmids were used as template DNA to prepare anti-sense or sense RNA probes. Whole-mount in situ hybridization was performed as previously described^24^.

**Table 1:** Primers used in preparation of probes.

Isoform	Primer	Sequence
a1	Va1Eco-S1	5′-CAGGAATTCTTGGTCACACCTTGGAGGAGGA-3′
	Va1Hind-A1	5′-CAGAAGCTTTTGGACAGGGTCCCCACAAAGAGCT-3′
a2	Va2-3UTR-S1	5′-GTTACAGAATTCACTCACTCACTC-3′
	Va2-3UTR-A1	5′-TTTGTAAGCTTGTCTCCGATTATTAAAAG-3′
a3	Va3Eco-S1	5′-TAGGAATTCCGGCGCTGCGAGGAACTGGAGAA-3′
	Va3Hind-A1	5′-CCTTAAGCTTGTGGATCTGCACCTGCCATGGG-3′
a4	Va4Eco-S1	5′-GGGGAATTCTGTCTGTATCCTGGTGCAGGCGA-3′
	Va4Hind-A1	5′-TATAAAGCTTGAAATACTGCAGGAACTTCAGG-3′

The PCR products were digested with restriction enzyme Hind III and EcoRI and then subcloned into pGEM11.

### Immunohistochemistry and fluorescence microscopy

Pregnant female mice (6- to 10-week-old) were anesthetized using isoflurane and perfused with a fixative solution (4% paraformaldehyde [PFA] in phosphate-buffered saline [PBS]). Embryos were dissected and fixed in 4% PFA. Embryos were staged according to the dissection time (noon of the vaginal plug as E0.5) and morphology. The fixed embryos were blocked in a blocking solution containing 0.05% Tween-20, 0.5% TSA blocking reagent (PerkinElmer), and 1% normal donkey serum in PBS, and then incubated with primary and secondary antibodies diluted in the blocking solution. Nuclear DNA was labelled with TO-PRO-3 (Thermo Fisher Scientific, USA). The same secondary antibody alone control experiments were also performed: specifically, Cy3-conjugated donkey anti-rabbit IgG was used for a1, a3, and a4, and Cy3-conjugated donkey anti-chick IgY for a2. The secondary antibody controls in which the embryos were labelled without primary antibodies against the a isoforms were carried out in parallel, and data were obtained under same image acquisition parameters, using same microscope. The images were processed with same adjustment scale using image processing systems.

Immunostained embryos were soaked in a 1:1 mixture of 40% glycerol and VECTASHIELD mounting medium (Vector Labs, USA), and then mounted in a solidified 0.1% gellan gum (Sigma-Aldrich, USA) prepared in PBS containing 40% glycerol and 0.05% Tween 20, and observed under a confocal laser scanning microscope (Zeiss LSM 510 or 800) as previously described^24,26^.

### Ethics approval

All applicable international, national, and/or institutional guidelines for the care and use of animals were followed. All animal procedures were approved by the Committees of Institute of Scientific and Industrial Research (ISIR), Osaka University (Dosan 27-1-0 and Dosan03-1-0), and Doshisha Women’s College of Liberal Arts (Y13-003 and Y21-008). This article does not contain any studies with human participants performed by any of the authors.

## Results

**Figure 1:**
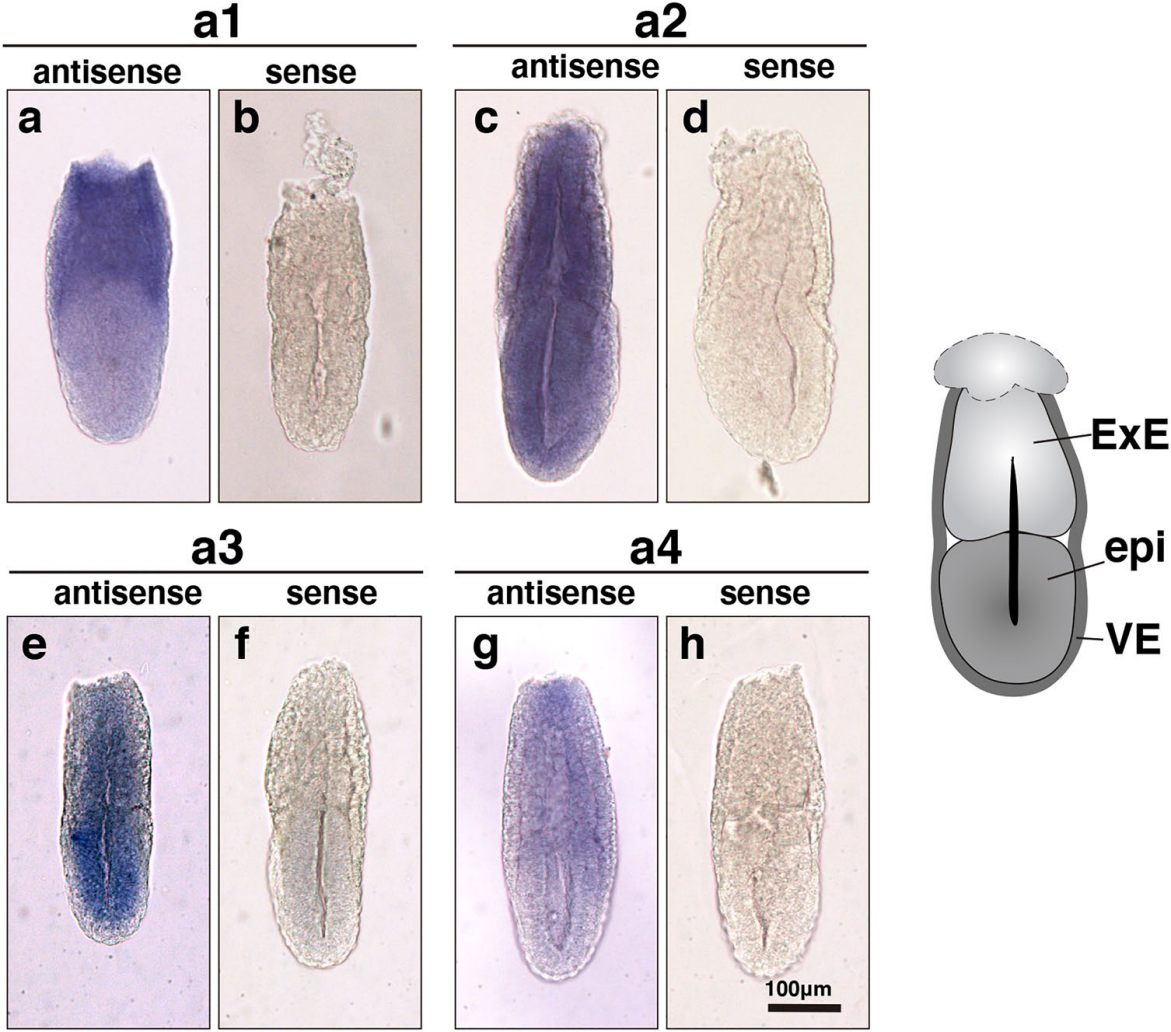
Whole-mount in situ hybridization analysis of a subunit isoform expression in mouse E6.5 embryos. Whole-mount in situ hybridization assays for a1, a2, a3, and a4 mRNA were carried out with anti-sense (**a**,**c**,**e**,**g**) or control sense (**b**,**d**,**f**,**h**) probes using E 6.5 mouse embryos. All assays were performed using at least three embryos. Specific labelling was observed with anti-sense probes, and no specific staining above background was observed with the negative control probes. An illustration of the E6.5 embryo is shown on the right. VE, visceral endoderm; ExE, extraembryonic ectoderm; epi, epiblast. Scale bar, 100 µm.

We first examined the transcripts of a subunit isoforms in embryos isolated at embryonic day 6.5 (E6.5) using whole-mount in situ hybridization with isoform-specific antisense probes. All the transcripts of the four isoforms were detected in E6.5 embryos (Fig. 1). The a1 mRNA was found in both the epiblast and extraembryonic ectoderm (ExE), and its expression level was higher in the ExE region (Fig. 1a,b). The a2 mRNA was distributed evenly in the E6.5 embryo (Fig. 1c,d). The a3 isoform exhibited higher expression in the epiblast region (Fig. 1e,f). The a4 isoform mRNA was expressed at a higher level in the ExE region (Fig. 1g,h). No signals were detected when a sense probe was used (Fig. 1b,d,f,h). In adult mice, a4 mRNA is highly expressed in the kidney and epididymis, and a4 targets the V-ATPase complex to the plasma membrane of renal intercalated cells and epididymal clear cells^12–15^. Subsequent studies have shown that the a4 isoform is not restricted to the renal tissue, but also in the epithelium like retinal pigmented epithelium (RPE) and epithelium in the choroid plexus. All these tissues function in the secretion of solutes and ions. However, it has been reported that the a4 isoform contains two alternative splicing variants, a4-I and a4-II, which differ in their first exon encoding the 5′-noncoding region. a4-II expression is not found in embryos, whereas a4-I expression is detected at developmental stages as early as embryonic day 7^33^. Despite the tissue-specific expression pattern of a4 at the adult stage, its expression at the E6.5 stage appeared to be even in both the ExE and epiblast (Fig. 1g).

**Figure 2:**
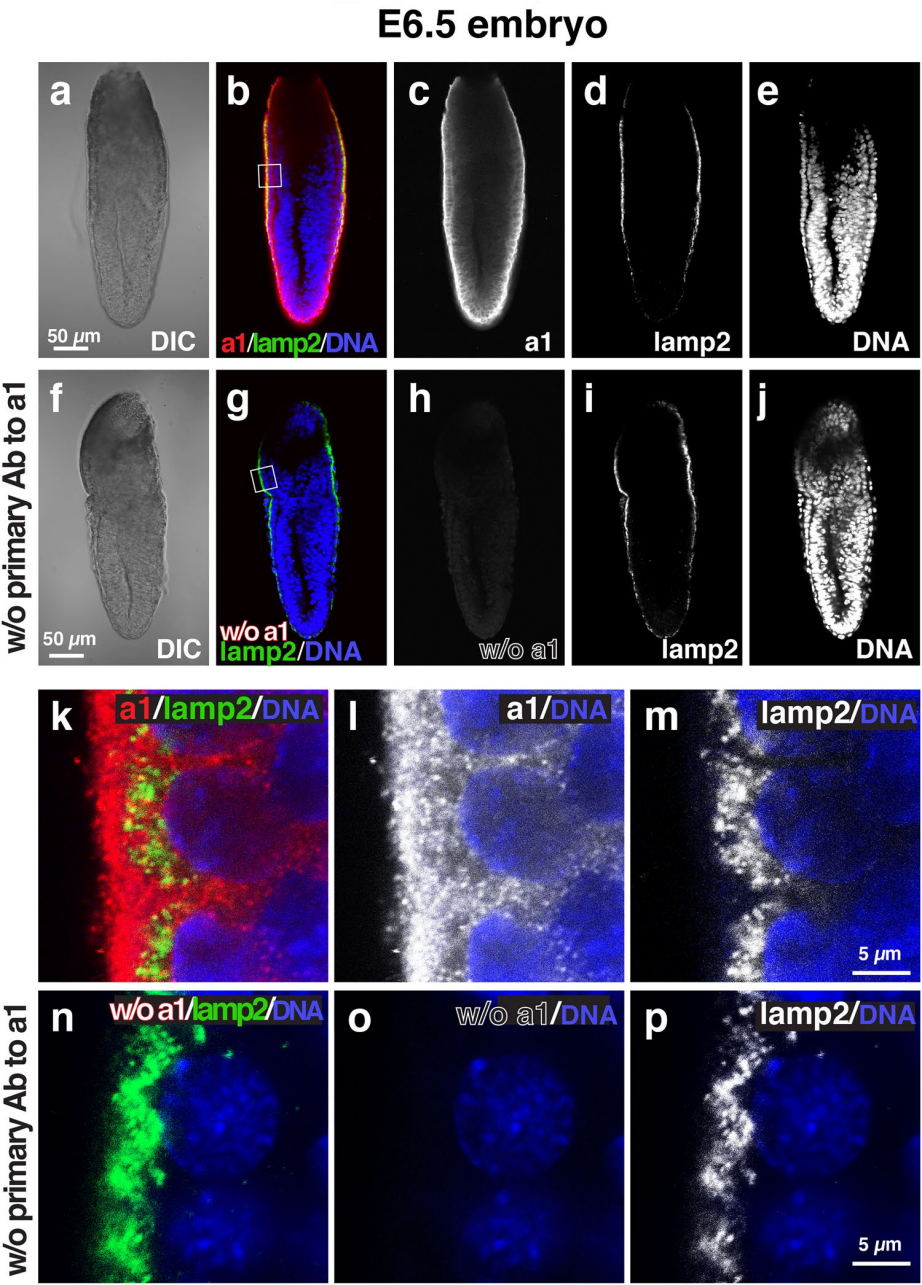
Localization of a1 subunit isoforms in mouse E6.5 embryos. Wild-type E6.5 embryos (n = 8) were isolated and stained with antibodies against a1 (red) and lamp2 (green). Nuclear DNA is also labelled (blue). The control embryos (n = 5) labelled without the primary antibodies against a1 (w/o primary Ab to a1) was shown in (**f**–**j**) and (**n**–**p**). The secondary antibody alone controls in which the embryos were labelled without primary antibodies against the a1 isoform were performed in parallel and the images were obtained under the same microscopic setup. The boxed region in (**b**,**g**) is magnified and shown as (**k**–**m**,**n**–**p**), respectively. Scale bars represent 50 µm and 5 µm in (**a**–**j**,**k**–**p**), respectively.

**Figure 3:**
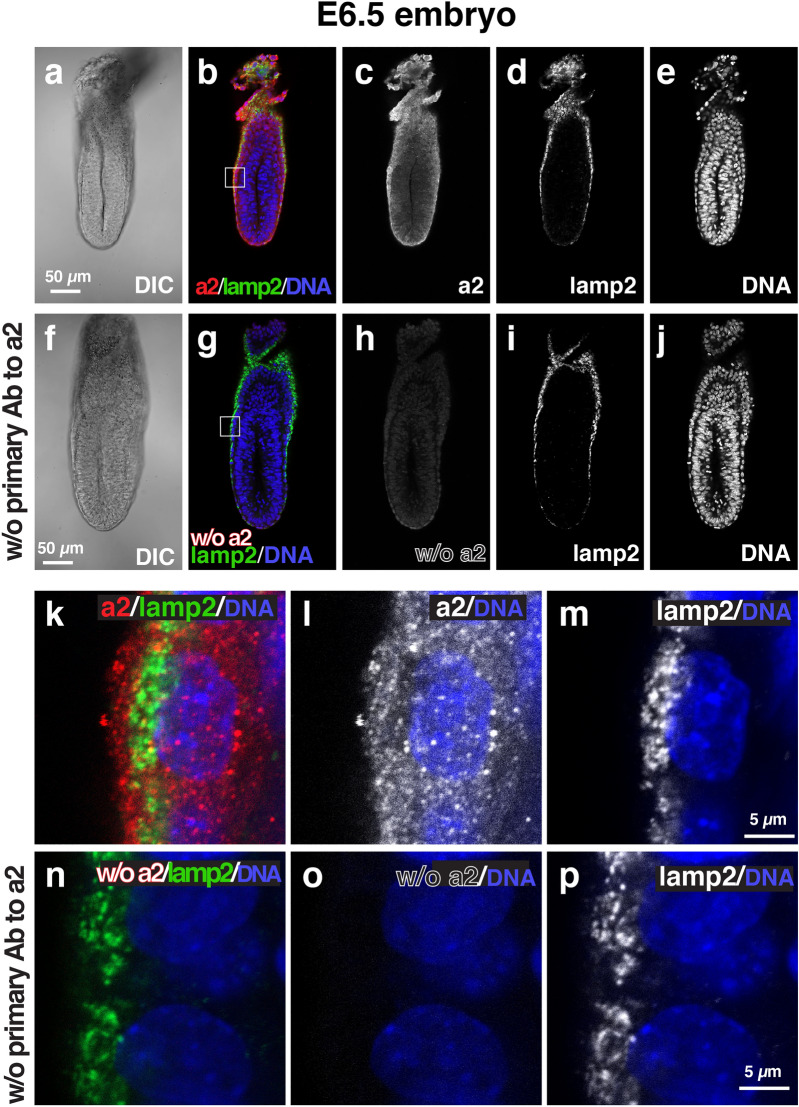
Localization of a2 subunit isoforms in mouse E6.5 embryos. Wild-type E6.5 embryos (n = 9) were isolated and stained with antibodies against a2 (red) and lamp2 (green). Nuclear DNA is also labelled (blue). The control embryos (n = 4) labelled without the primary antibody against a2 (w/o primary Ab to a2) was shown in (**f**–**j**,**n**–**p**). The secondary antibody alone controls in which the embryos were labelled without primary antibodies against the a2 isoform were performed in parallel and the images were obtained under the same microscopic setup. The boxed region in (**b**,**g**) is magnified and shown as (**k**–**m**,**n**–**p**), respectively. Scale bars represent 50 µm and 5 µm in (**a**–**j**,**k**–**p**), respectively.

We then examined the localization of each isoform using immunofluorescence microscopy with isoform-specific antibodies^18,29^. The immunofluorescence signals for a1 (Fig. 2) were higher in the VE cells than in the epiblast (Fig. 2b,c). The intracellular localization of a1 in VE cells was mainly detected in both the apical and basolateral regions (Fig. 2k,l). In the VE of E6.5 embryos, lamp2, a lysosomal membrane protein, was used as a marker of apical vacuoles^24–26^. Lamp2 showed a ring-like staining pattern by staining the limiting membranes of the apical vacuoles (Fig. 2h). However, a1 signals did not colocalize with lamp2 (Fig. 2k). The secondary antibody alone controls confirmed that signals for a1 were considerably low when the embryos were labelled without the primary antibodies against a1 (Fig. 2f–j,n–p). The immunostaining signals for a2 were observed in both VE cells and epiblasts (Fig. 3). The signals in the epiblast appeared higher than those observed in the VE cell. The a2 signals did not overlap with those of lamp2 (Fig. 3f), suggesting that a2 is not associated with the apical vacuole. Rather, they were observed as a dot-like pattern in the apical region of VE cells (Fig. 3k,I), where the endocytic vesicles are highly developed, reflecting active endocytosis by the VE cells^23–25,34^. In the absence of the primary antibody to a2, the signals were found to be considerably low under the same detection conditions (Fig. 3f–j,n–p). The a2 subunit is known to be associated to the Golgi apparatus^18,35,36^. Confirming this subcellular location, its loss of function brings defects in glycosylation of cell surface proteins^37,38^. In renal epithelial cells, a2 is also a component of early endosomes^21^. In the embryonic epithelium, we showed that a2 is enriched in the apical cytoplasm of VE cells, where numerous endocytic vesicles are present. This observation reinforces the previous result showing a2 expression in early endosomes^21^.

**Figure 4:**
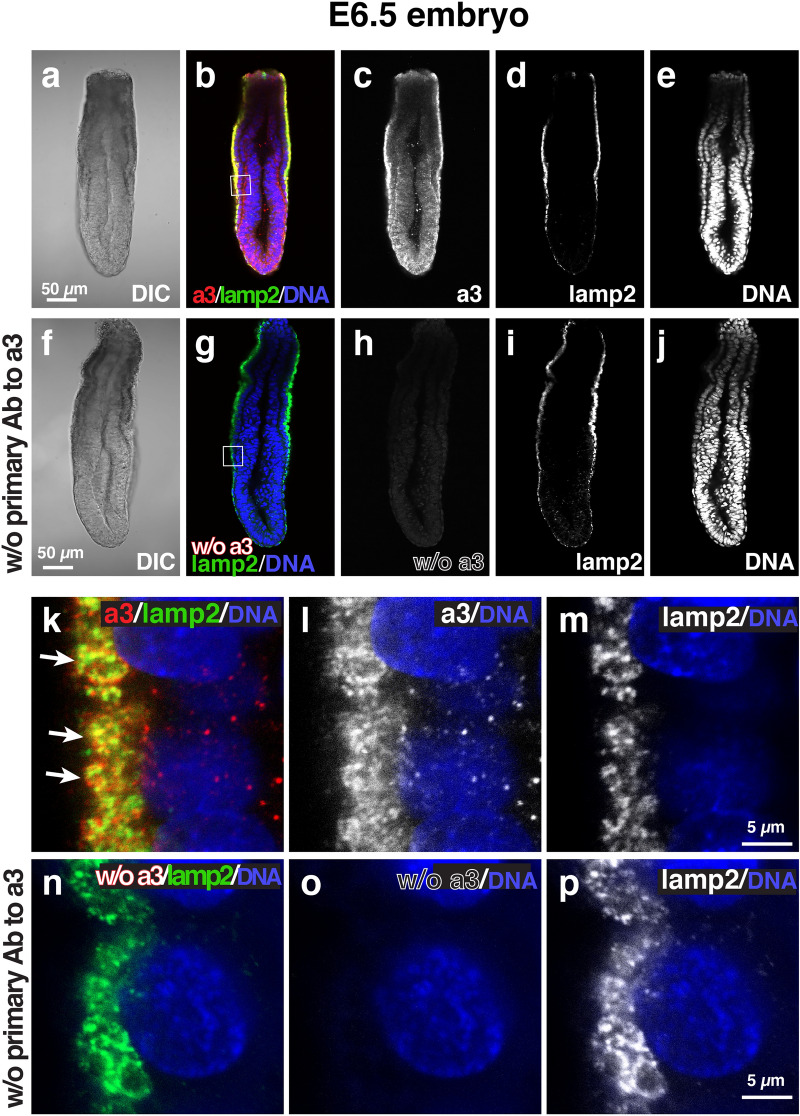
Localization of a3 subunit isoforms in mouse E6.5 embryos. Wild-type E6.5 embryos (n = 10) were isolated and stained with antibodies against a3 (red) and lamp2 (green). Nuclear DNA is also labelled (blue). Arrows indicate the apical vacuole. The control embryos (n = 5) labelled without the primary antibodies against a3 (w/o primary Ab to a3) was shown in (**k**–**j**,**n**–**p**). The secondary antibody alone controls in which the embryos were labelled without primary antibodies against the a3 isoform were performed in parallel and the images were obtained under the same microscopic setup. The boxed region in (**b**,**g**) is magnified and shown as (**k**–**m**,**n**–**p**), respectively. Scale bars represent 50 µm and 5 µm in (**a**–**j,k**–**p**), respectively.

The immunofluorescence signals for a3 were more concentrated in VE than in epiblasts (Fig. 4). In the epiblast, a3 exhibited dot-like staining patterns, as shown in Fig. 4b,c. In the VE, the staining pattern of a3 resembled that of lamp2 (Fig. 4b–d). At E6.5, cells in the VE layer are not uniform in morphology or molecular characteristics^23^. VE cells covering the epiblast (embryonic VE) are a group of monolayer cells with squamous morphology, while those concealing the extra-embryonic VE (ExE) are cuboidal. Extra-embryonic VE cells contain lamp2-positive large apical vacuoles (Fig. 4d), as described previously^24,25^. The signals of a3 overlapped well with those of lamp2 (Fig. 4b,k,l,m, arrows). In the absence of the primary antibody to a3, the apical vacuole signals were not detectable under the same detection conditions (Fig. 4f–j,n–p). These results suggest that the a subunit isoform localized on the apical vacuole membrane is a3.

**Figure 5:**
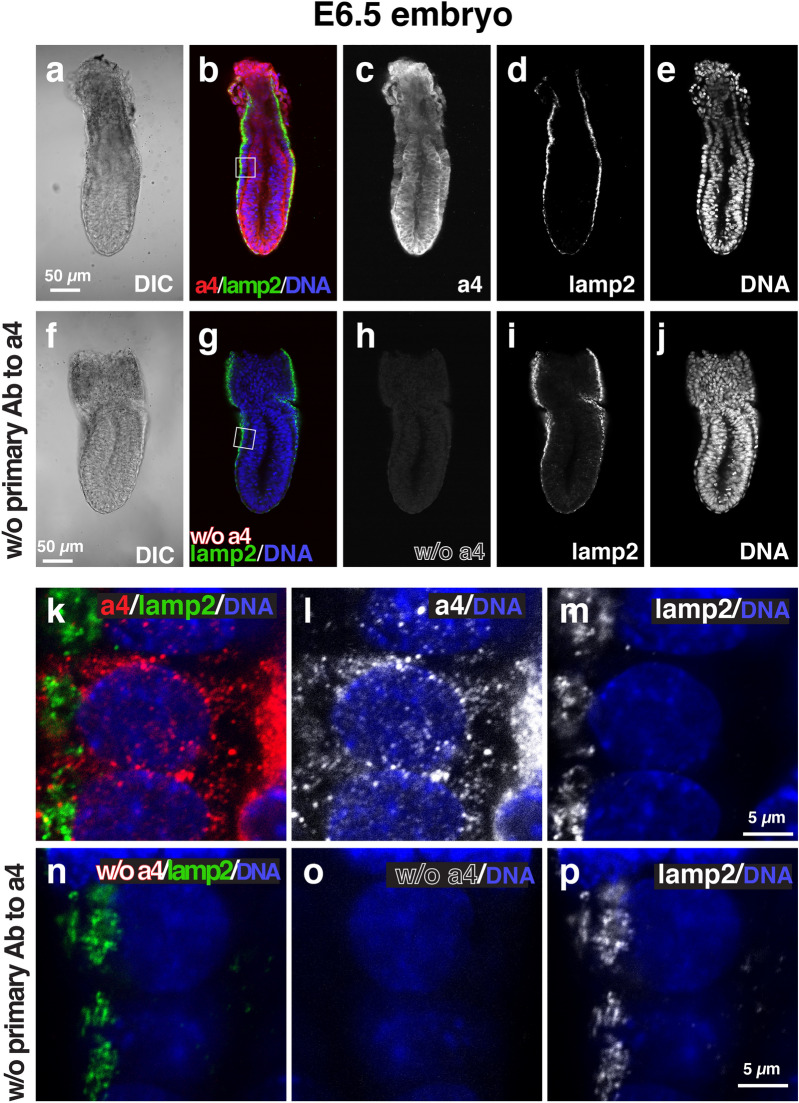
Localization of a4 subunit isoforms in mouse E6.5 embryos. Wild-type E6.5 embryos (n = 8) were isolated and stained with antibodies against a4 (red) and lamp2 (green). Nuclear DNA is also labelled (blue). The control embryos (n = 5) labelled without the primary antibodies against a4 (w/o primary Ab to a4) was shown in (**k**–**j,n**–**p**). The secondary antibody alone controls in which the embryos were labelled without primary antibodies against the a4 isoform were performed in parallel and the images were obtained under the same microscopic setup. The boxed region in (**b**,**g**) is magnified and shown as (**k**–**m**,**n**–**p**), respectively. Scale bars represent 50 µm and 5 µm in (**a**–**j**,**k**–**p**), respectively.

The a4 signals were observed in both VE and epiblasts, although the signals in VE were lower than those in epiblasts (Fig. 5). The distribution was consistent with the mRNA expression pattern shown in Fig. 1 (panels g and h). The signals of a4 were found in the perinuclear regions of epiblast cells and were found as dot-like signals in VE cells (Fig. 5k,l). The distribution of a4 in adult tissue is frequently observed in apical or basolateral plasma membranes^12,15,30^; it is considered to be responsible for extracellular acidification. The localization patterns of the a4 subunit in VE cells were distinct from those in the other epithelial tissues. In the absence of the primary antibody to a4, we could not detect any signals under the same image acquisition conditions (Fig. 5f–j,n–p).

## Discussion

Intracellular luminal acidification is essential for various cellular processes, including vesicular trafficking, solute transport, neurotransmitter loading, and degradation of macromolecules. These processes are fundamental for the establishment and maintenance of functional integrity in multicellular organisms. We have shown that acidification driven by V-ATPase is required for the early development of mouse embryos^22,39^. We found that genetic disruption of the V-ATPase function resulted in loss of apical-basolateral polarity in the embryonic epithelium (visceral endoderm, VE) before the gastrulation stage and caused embryonic lethality soon after implantation. The apical vacuole, a unique lysosomal compartment in VE cells, shares characteristics with ‘normal’ lysosomes^40^. We found that the apical vacuole was acidified, although the most prominent acidic compartment in VE cells were early endosomes in the subapical cytosol^22^. This finding led us to examine which a subunit isoforms are responsible for maintaining the luminal acidification in embryonic tissues, because the a subunit isoforms are primarily important for determining the subcellular location as well as function of V-ATPase of various compositions^10,41^. In addition, the luminal acidic apical vacuole of visceral endoderm is important not only in nutrient uptake but also in controlling molecular signaling and developmental patterning^24–28^. We focused on which a isoform would localized on the apical vacuole.

The a1 isoform exhibited subapical localization in VE cells, similar to the c subunit^22^. The expression pattern was consistent with the phenotype of the a1 targeting mutation. The development of a1-knockout mice stopped at E5–6 before gastrulation^42^, thus the a1 function is indispensable for early development. Lethality occurs at the same developmental stage, where the loss of the c-subunit results in^22,39^. As discussed above, no isoform has been identified for the c subunit so far in mammals, and this proteolipid is the essential component for all V-ATPases^43^, thus, V-ATPase with the a1 subunit isoform plays a principally important role even in the earliest stage of morphogenesis. Subcellular and tissue defects associated with the loss of the a1 subunit isoform are therefore of great interest.

The a2 subunit isoforms are localized to the Golgi apparatus and early endosomes^18,21,36^. Genetic defects in the a2-encoding gene *ATP6V0a2* develop cutis laxa type IIa or wrinkly skin syndrome in affected humans. Fibroblasts lacking the a2 isoform show dysfunction of Golgi assembly, *N*- and *O*-linked glycosylation, and secretion and assembly of extracellular matrix proteins, including tropoelastin^37,38^. In mice, a2 function has been implicated in the assembly of extracellular matrix^44^ and cytotoxic T-cell differentiation^45^ as well as transduction of tumour growth factor beta and Notch/Delta signalling^46^. It has been reported that the expression of V-ATPase with the a2 isoform is upregulated in preimplantation embryos^47^. Its function is not an absolute requirement for early embryogenesis, because no phenotypes in early development have been reported to date. In this study, however, we showed that the a2 subunit isoform is expressed and accumulated in the perinuclear regions of the visceral endoderm and in the cytoplasm of the distal ExE and proximal epiblast. Importantly, these embryonic tissues are highly active in the synthesis and degradation of multiple signalling molecules, including FGF/heparan sulfate proteoglycans^48,49^ and the *O*-glycosylated protein Cripto, an extracellular component of tumour growth factor beta/Nodal signalling^50^. The lack of phenotypic difference in embryogenesis does not extenuate the significance of this subunit isoform. Various studies have revealed that functional redundancy among the subunit isoforms could mask the development of apparent defects in various cases^36,51,52^.

Here, we showed that the a3 isoform is localized at the membranes of the apical vacuole. Previously, we generated mice carrying a deletion mutation in *Tcirg1*, the gene encoding the a3 isoform^35^. The a3-deficient mutant mice were indistinguishable at birth; however, by two weeks of age, they were smaller than their normal littermates. They could not survive for more than a month and showed various defects, including in bone remodelling, hormone secretion, and retinal degeneration^11^. However, no obvious phenotype was observed during the gastrulation stage. It is possible that other isoforms may replace a3 at pre-gastrulation stage. We found that the expression of the a2 isoform is upregulated and a2 compensates for the function of a3 in pancreatic β cells^36^. The a3 function in osteoclasts could be replaced partially by overexpression of a1 or a2^32^. It is possible that a combination of multiple isoforms that target mutations may cause defects in early development.

Previous observations revealed that the splicing variant a4-I of the a4 isoform was detected at embryonic day 7^33^. This is consistent with our finding that the a4 isoform is highly expressed in embryonic tissues. Genetic lesions in human *ATP6V0A4*, encoding the a4 subunit, develop severe dysfunction in acid/base homeostasis due to defective renal acidification^53^. In mice, loss of function of *Atp6v0a4* allows the pups to develop to term and deliver, although they suffer severe renal dysfunction and hearing loss; however, to the best of our knowledge, no embryonic phenotype has been reported. The lack of an apparent phenotype at the peri-gastrulation stage may reflect that other V-ATPase subunit isoforms would provide enough support to compensate for the loss of a4. Our finding that the a4 isoform is truly expressed in the early stage of development means that this subunit isoform should be taken into account for the interpretation of embryonic phenotypes given by single and multiple lesions of the V-ATPase a isoforms.

Overall, the differential localization of a subunit isoforms of V-ATPase in mouse peri-gastrulation embryos may implicate the cell- and organelle-specific requirements of the enzyme. These results provide useful information for further investigation of the involvement of specific steps in the endocytic pathway in early embryonic development.

## Data Availability

The datasets used and/or analysed during the current study available from the corresponding author on reasonable request.
